# Supplementary material for: Protocol for the ‘exploration of mental well-being, health-related quality of life, and psychosocial outcomes in youths on dialysis in New Zealand: A cross-sectional methods study’
Source: PLoS One. 2026 Feb 19;21(2):e0340006. doi: 10.1371/journal.pone.0340006 (PMC12919795; doi:10.1371/journal.pone.0340006)
Supplement: Attachment — Submitted filename: PONE-D-25-52324 Review TB.docx [file pone.0340006.s001.docx]

Manuscript Number: PONE-D-25-52324

Title: Protocol for the 'Exploration of Mental Well-Being, Health-Related Quality of Life, and Psychosocial Outcomes in Youths on Dialysis in New Zealand: A Cross-Sectional Methods Study'

**General Comments**

This manuscript outlines a study protocol aimed at investigating the physical and psychosocial health needs of youths (aged 15–24) undergoing dialysis in Aotearoa New Zealand (AoNZ). The study plans to employ a cross-sectional survey design using a wide variety of validated psychometric instruments to assess mental well-being, quality of life (QOL), self-efficacy, and social anxiety, alongside clinical data.

General Interest and Relevance

The subject matter is of significant interest to the nephrology community. As the authors note, youths on dialysis represent a vulnerable population facing specific challenges distinct from older adults. The medical community is constantly seeking precise, personalized, and reproducible approaches to Patient-Reported Outcomes (PROs), making this data highly relevant. The attempt to capture the "unmet needs" in this transition-age group is commendable.

However, while the objective is clear, the methodology presents significant concerns regarding statistical power and the validity of the proposed analysis plan. The study aims to recruit from a total estimated population of only 50 patients, with an expected sample size of 25–30 participants^5^. The proposal to use Structural Equation Modeling (SEM) on such a small sample, combined with a proliferation of different scales, introduces a high risk of statistical bias and overfitting.

**Major Issues**

1. Statistical Methodology and Sample Size Constraints

The most critical weakness of this protocol is the disconnect between the sample size and the proposed analytical complexity.

- **Sample Size:** The authors anticipate a recruitment of 25–30 participants^6^.
- **Analytical Plan:** The authors propose using Structural Equation Modeling (SEM) to explore associations between psychometric constructs^777^.
- **Critique:** SEM is a large-sample technique. A general rule of thumb suggests a minimum of 10–20 subjects per estimated parameter, or absolute sample sizes often exceeding 100–200. Applying SEM to a sample of $N \approx 30$ is statistically invalid and likely to yield unstable, non-reproducible results (statistical bias).
- **Recommendation:** The authors state they have not explored clustering analyses. Given the semi-quantitative nature of the scores (Likert scales, frequency scores), a cluster analysis approach would be far more robust and appropriate for this sample size. Instead of trying to model complex causal pathways (which requires high power), the study should aim to identify *profiles* or *clusters* of patients sharing similar psychosocial characteristics. This would provide actionable clinical phenotypes without overextending the data.

2. Multiplication of Scales vs. Patient Burden

The study proposes a very high number of instruments for a single survey:

- SWEMWBS (Mental well-being) ^8^
- EQ-5D VAS & EQ-5D-3L (HRQoL) ^9999^
- PedsQL 3.0 ESRD (Disease-specific QOL) ^10^
- Hua Oranga (Māori health) ^11^
- ADL scales (Activities of Daily Living) ^12^
- RAPA (Physical activity) ^13^
- GSE (Self-efficacy) ^14^
- SAD-D (Social anxiety) ^15^
- CES-DC (Depression) ^16^
- Health Literacy screener ^17^

While comprehensive, multiplying scales for such a small number of patients increases the risk of Type I errors (multiple testing) if not corrected, and increases respondent burden. The authors should justify why this specific combination is necessary versus a more streamlined approach, or explicitly state how they will control for multiple comparisons in their descriptive statistics.

3. The "Transition" Dilemma: Pediatric vs. Adult Scales

The study justifies the use of both adult (e.g., RAPA, EQ-5D) and pediatric (e.g., PedsQL, CES-DC) scales. This highlights the unique nature of the 15–24 age group transitioning between pediatric and adult medicine.

- **Strength:** It attempts to bridge the gap.
- **Weakness:** It creates methodological heterogeneity. Comparing results from this cohort to pure adult or pure pediatric cohorts in the literature will be difficult. The authors need to discuss the validity of using the RAPA (validated for older adults) in 15-year-olds more critically^18^, despite their pilot testing.

4. Cultural Validity

Nearly half of the target population belongs to Māori and Pacific ethnic groups19. This raises valid questions about the suitability of standard Western tools (like the CES-DC or GSE) for approaching their mental well-being.

- The inclusion of the *Hua Oranga* tool is a major strength and a necessary addition^20^.
- However, the protocol should discuss whether the *other* chosen tools (PedsQL, SWEMWBS) have been validated or culturally adapted for Māori youth. If not, this limitation must be acknowledged as a potential source of measurement bias.

5. Lack of Critical Assessment of Existing Framework

The protocol provides a detailed list of tools but lacks a critical stance regarding published dialysis trials on this topic. Specifically, the PROMIS (Patient-Reported Outcomes Measurement Information System) scales are increasingly the standard in nephrology research for assessing physical, mental, and social health (CONVINCE PROMs results).

- Why were the selected legacy instruments chosen over PROMIS measures?
- A discussion comparing their chosen methodology to the PROMIS framework is expected to justify the selection and ensure the data is comparable with modern international registries.

**Specific Recommendations**

1. **Revise the Analysis Plan:** Remove the intention to perform Structural Equation Modeling (SEM)^21^. It is methodologically unsound for $N=30$. Replace it with a plan for descriptive statistics and **Cluster Analysis** (or Latent Class Analysis adapted for small samples) to identify patient typologies based on the semi-quantitative scores.
2. **Address Statistical Power:** Explicitly acknowledge the limitations of the sample size regarding inferential statistics.
3. **Justify Tool Selection:** Add a paragraph comparing the chosen tools against the PROMIS measures. Explain the decision-making process (e.g., is it to maintain longitudinal continuity with local historical data?).
4. **Cultural Validation:** Expand on the validity of the non-indigenous scales (like RAPA and SAD-D) for Māori and Pacific youth.

**Conclusion**

**This study addresses a vital gap in the care of youths on dialysis. The data will be highly relevant to the nephrology community. However, the current statistical plan (SEM) is a significant weakness given the limited patient population. Shifting the analytical focus toward clustering and descriptive profiling would make the protocol scientifically robust.**

**Nevertheless, there is a real need and benefit to collecting comprehensive structured data within this unstudied population and *PLOS ONE* should support the proposal.**

***Note to the Editor:* If this methodology paper is accepted, *PLOS ONE* should be the venue for the results of the study to ensure the complete research cycle is available in one place.**

**Recommendation:** Minor Revision
