# Supplementary material for: Protocol for the ‘exploration of mental well-being, health-related quality of life, and psychosocial outcomes in youths on dialysis in New Zealand: A cross-sectional methods study’
Source: PLoS One. 2026 Feb 19;21(2):e0340006. doi: 10.1371/journal.pone.0340006 (PMC12919795; doi:10.1371/journal.pone.0340006)
Supplement: Attachment — Submitted filename: Response to reviewers.docx [file pone.0340006.s003.docx]

Hansani Madushika Abeywickrama, Ph.D.
Academic Editor
PLOS ONE

Dear Dr Abeywickrama

**Response to Reviewers**

***Journal Requirements:***

When submitting your revision, we need you to address these additional requirements.

1. Please ensure that your manuscript meets PLOS ONE's style requirements, including those for file naming. The PLOS ONE style templates can be found at

<https://journals.plos.org/plosone/s/file?id=wjVg/PLOSOne_formatting_sample_main_body.pdf> and

<https://journals.plos.org/plosone/s/file?id=ba62/PLOSOne_formatting_sample_title_authors_affiliations.pdf>

Thank you, we have made changes in style to comply with requirements.

2. Thank you for stating the following financial disclosure:

“University of Auckland Open Access Grant as part of general PhD funding”

Please state what role the funders took in the study.  If the funders had no role, please state: "The funders had no role in study design, data collection and analysis, decision to publish, or preparation of the manuscript."

Thanks you we have done this.

3. We note that you have indicated that there are restrictions to data sharing for this study. PLOS only allows data to be available upon request if there are legal or ethical restrictions on sharing data publicly. For more information on unacceptable data access restrictions, please see <http://journals.plos.org/plosone/s/data-availability#loc-unacceptable-data-access-restrictions>.

Before we proceed with your manuscript, please address the following prompts:

a) If there are ethical or legal restrictions on sharing a de-identified data set, please explain them in detail (e.g., data contain potentially identifying or sensitive patient information, data are owned by a third-party organization, etc.) and who has imposed them (e.g., a Research Ethics Committee or Institutional Review Board, etc.). Please also provide contact information for a data access committee, ethics committee, or other institutional body to which data requests may be sent.

b) If there are no restrictions, please upload the minimal anonymized data set necessary to replicate your study findings to a stable, public repository and provide us with the relevant URLs, DOIs, or accession numbers. For a list of recommended repositories, please see

<https://journals.plos.org/plosone/s/recommended-repositories>. You also have the option of uploading the data as Supporting Information files, but we would recommend depositing data directly to a data repository if possible.

We will update your Data Availability statement on your behalf to reflect the information you provide.

Thank you. We apricate that the ambiguity in "reasonable request" has been criticized in open science discussions, and that many advocate for clearer data sharing commitments like public repository deposits to avoid gatekeeping. We have clarified the restrictions on data sharing, which we have expanded upon on page 17 of the manuscript. Of note, some of these restrictions are legal. See below:

#############################START##############################

Authors agree to make data and materials supporting the results or analyses presented in their paper available upon reasonable request. It is up to the authors to determine whether a request is reasonable. Several factors will be considered in evaluating requests:

•	Legitimacy of scientific purpose: requests will be viewed more favorably if the intention is replication, meta-analysis, secondary analysis, or educational use.

•	Requester credentials: requests from researchers at recognized institutions with relevant expertise will be viewed more favorably.

•	Feasibility and burden: reasonable requests should not impose excessive work on the authors

•	Ethical and legal constraints: certain data containing sensitive information have restrictions on sharing, as specified in data use agreements and ethics / IRB approvals. In particular, as a matter of national policy in Aotearoa New Zealand, there is a relative prohibition around sending Māori research data overseas consistent with the principles of Māori self-determination and control over data about their communities, rooted in Te Tiriti o Waitangi (the Treaty of Waitangi) and as specified in the (NZ) Data and Statistics Act 2022. Sharing of such data would require an evaluation from the national (NZ) Health and Disability Ethics Committee and as well as IRBs of all the participating hospitals, who will decide whether it is possible enforce NZ rules and norms pertaining to such data, including but not limited to: (1) whether the overseas jurisdictions have comparable data protection safeguards to NZ; (2) whether there will be culturally appropriate representation on overseas governance committees to ensure that the data are handled in culturally appropriate ways with a good understanding of the NZ Māori community that the data relate to

•	Specificity: only specific requests for variables relevant to well-defined research question will be considered

•	Timing: requests made shortly after publication will be considered whereas those made a substantial amount of time later may not be possible when authors have moved on or data storage might have lapsed.

#############################FINISH##############################

4. Please include captions for your Supporting Information files at the end of your manuscript, and update any in-text citations to match accordingly. Please see our Supporting Information guidelines for more information: [http://journals.plos.org/plosone/s/supporting-information](http://track.editorialmanager.com/CL0/http:%2F%2Fjournals.plos.org%2Fplosone%2Fs%2Fsupporting-information/1/010f019abf2834c2-2e756baf-69fd-45ae-b196-75aba6d73657-000000/l8ea0d6YnqeIEzxpACN-o2sYDIoye6QITX7Fq2Rqv9I=238).

N/A

5. If the reviewer comments include a recommendation to cite specific previously published works, please review and evaluate these publications to determine whether they are relevant and should be cited. There is no requirement to cite these works unless the editor has indicated otherwise. 

N/A

6. Please review your reference list to ensure that it is complete and correct. If you have cited papers that have been retracted, please include the rationale for doing so in the manuscript text, or remove these references and replace them with relevant current references. Any changes to the reference list should be mentioned in the rebuttal letter that accompanies your revised manuscript. If you need to cite a retracted article, indicate the article’s retracted status in the References list and also include a citation and full reference for the retraction notice.

Thank you, and checked

***Reviewer #1comments:***

The current statistical plan (SEM) is a significant weakness given the limited patient population. Shifting the analytical focus toward clustering and descriptive profiling would make the protocol scientifically robust.

Thank you, this is a very useful and helpful comment.

Firstly, we think a structural / causal analysis is warranted to create actionable insights with a likely benefit in idealistically a deterministic manner (but of course a practically stochastic one). We have been clear that we are using PLS-SEM rather than CB-SEM. The former is very forgiving to smaller datasets and would allow reasonable statistical inference in this sample, in our estimation. The corresponding author has several publications using this methodology in samples with n < 100, and is confident of being able to obtain at least a signal of findings if they are there to be found. The overall chance of model convergence and statistical significance is hampered by the small sample size, of course, but this leads to Type II rather than Type I errors. The group is happy to work within this constraint.

The suggestion around cluster analysis +/- hierarchical clustering with dendrograms +/- decision tree methods like CHAID or CART etc is a great idea, although we did not specify this analysis in our statistical analysis plan, let alone details such as the number of clusters in advance. This will be a great post hoc study and we might include latent class analysis as well, in case there are subgroups that we cannot identify a priori, but which become clear based on patterns of survey responses. Once we have those clusters mapped, we will determine how demographic and behavioral responses differ across them. Such an approach might create further actionable insights within discrete respondent groups in our sample.

We have added a statement of intent around these extended analyses in the discussion on Page 15.

***Reviewer #1comments:***

The word 'youth' throughout sometimes needs to be plural wonder if young adult or young adults might be better

Thank you. We too have struggled with this at times. “Youth" can be a singular noun, referring to one young person. It can also be used collectively for a group of young people, as an uncountable noun. We have gone through the paper and made sure that “youth” is used as a singular noun or used collectively, and made sure that we have used the term “youths” for more than one person who all happen to fit the conceptual definition.

Speaking of which, "youth" is an official demographic term. For statistical and research purposes, the UN defines 'youth' as persons between ages 15 and 24, a definition established in 1981 during preparations for the International Youth Year. All UN statistics on youth are based on this definition, as reflected in annual yearbooks on demography, education, employment, and health United Nations. WHO defines adolescents as individuals between 10 and 19 years, young people as those aged 10-24, and youth as those aged 15-24.

We feel that we should be clear about which definition of younger persons we are studying, and introducing the term “young adults” we think would create confusion.

The PPIE section there is sentence started and not completed

Thank you, this error has been corrected.

Just some clarity on exclusion criteria as further on you state has a 'documented cognitive or physical impairment that prevents self-completion, assistance from a parent or caregiver will be permitted'- just need to be clear why is this different to what is described as severe in the exclusion criteria

Thank you for this. The issue is not really one that pertains to communication difficulties, but rather pertains to those patients with severe functional difficulties or disabilities that are not appropriate for assessment by the survey instrument. The verbiage in the paper was carried through from the protocol and was clearly an error. We have corrected this in the manuscript on page 7.

An extensive list of outcome measures including clinical variables just a question as this is for people on dialysis have you considered any questions on their role within their dialysis treatment relating to self efficacy?

Thank you for this great suggestion. We had not though of this, and will triangulate and correlate GSES with dependent versus independent modalities of dialysis. This maybe an additional paper and would suit a clustering and descriptive profiling paper which we should do as a post-hoc exercise. We have added statement of intent around these extended analyses in the discussion on Page 15-16.
